# Use of Artificial Intelligence in Difficult Airway Assessment: The Current State of Knowledge

**DOI:** 10.3390/jcm14051602

**Published:** 2025-02-27

**Authors:** Mateusz Wilk, Wojciech Pikiewicz, Krzysztof Florczak, Dawid Jakóbczak

**Affiliations:** 1 Collegium Medicum, WSB University, 41-300 Dabrowa Gornicza, Poland; wojciech.pikiewicz@wsb.edu.pl; 2 Emergency Medical Centre in Opole Adama Mickiewicza 2, 45-367 Opole, Poland; krysmedflorczak@gmail.com (K.F.); jakobczakmedic@gmail.com (D.J.)

**Keywords:** artificial intelligence, airways, difficult, assessment

## Abstract

Artificial Intelligence (AI) has become one of the most transformative technologies of the 21st century. It is poised to reshape medicine, as almost every field of hospital treatment has seen an increase in AI’s presence. In this article, we focus on its impact in the field of anesthesia. We discuss its possible influence on difficult airway management, as it remains one of the most critical and potentially hazardous aspects of anesthesia, often leading to life-threatening complications. The accurate prediction of difficult airways can significantly improve patient safety. We covered the available literature on AI-based models for difficult airway prediction in comparison to traditional forms of airway assessment, as well as the predictive value of ultrasonography. We also address the narrative that AI-based algorithms show high sensitivity and specificity, with which they significantly outperform classical tests based on complex scales and indices.

## 1. Introduction

Difficult airway management remains one of the most important and potentially hazardous aspects of anesthesia, sometimes leading to life-threatening complications if not detected early enough and then properly managed. The accurate prediction of difficult airways can significantly improve patient safety by enabling clinicians to take appropriate preparatory measures before anesthesia induction [1]. Traditional airway assessment methods, such as the Mallampati score, the Cormack-Lehane classification, and physical examination, have been widely used to predict difficult intubation. However, these methods have inherent limitations, including subjectivity, lack of sensitivity, and poor reproducibility. Recent advancements in artificial intelligence (AI), particularly in machine learning (ML) and deep learning (DL), offer promising solutions by leveraging large datasets and computational models to improve the accuracy and reliability of airway predictions.

Almost every field of hospital treatment has seen an increase in AI’s presence with satisfactory results, based on more and more new research and publications [2]. AI used in medicine evolved from the field of telemedicine. The components used in it, very often combine both the sending of video and audio, as well as specific measurable parameters of the patient’s condition [3].

Machine learning (ML) utilizes sophisticated mathematical models and algorithms to analyze large datasets and extract meaningful insights without human intervention. During the training process, an ML algorithm is optimized to recognize specific patterns or generate outputs based on the given task. The result of this training is a machine learning model, typically implemented as a computer program with defined rules and data structures.

ML models are capable of identifying patterns and making predictions on previously unseen data. They employ various techniques to perform artificial intelligence (AI) tasks, including natural language processing (NLP), image recognition, and predictive analytics. In NLP, ML models can interpret and classify the intent behind textual input, while in image recognition, they can learn to detect and categorize objects, such as vehicles or animals.

One of the most common types of ML is supervised learning, where the algorithm is trained on a labeled dataset, allowing it to make predictions based on new, unseen data. Another type is unsupervised learning, which deals with data that has no labels and aims to uncover hidden patterns or relationships [4].

Deep learning, a specialized area within machine learning, leverages neural networks with multiple layers (also known as deep neural networks). These networks are inspired by the structure of the human brain, where neurons are connected in layers that process and transmit information. In AI systems, neural networks are trained by adjusting the weights of connections between neurons to minimize errors in prediction [5].

A particularly successful type of neural network is the convolutional neural network (CNN), which is widely used in computer vision tasks. CNNs use convolutional layers to automatically detect features such as edges, textures, and shapes, making them highly effective for image recognition and classification tasks. Convolutional neural networks (CNNs) distinguish themselves from traditional machine learning algorithms by their capability in automatically extracting features on a large scale, thereby eliminating the need for manual feature engineering [6].

Another breakthrough in AI is the use of recurrent neural networks (RNNs), which are particularly useful for sequential data such as time series or natural language. Long Short-Term Memory (LSTM) networks, a type of RNN, are designed to remember long-term dependencies, making them highly successful in applications like speech recognition and language translation [7].

### 1.1. Difficult Intubation and Its Frequency

The endotracheal intubation procedure, especially involving a difficult or compromised airway, is performed throughout the world, across the spectrum of hospital treatment, prehospital medicine and in the training of medical staff. It is inevitably associated with the use of bougie guidewires, differentiated laryngoscopic blades or videolaryngoscopes and knowledge of pharmacotherapy to facilitate visualization of the airway [8]. In the available literature, definitions of difficult intubation or difficult airway intubation are variable and depend on the country creating specific procedures or local protocols or guidelines. The American Society of Anesthesiologists, defines a difficult airway when “the anesthesiologist has difficulty ventilating the upper airway through a face mask, difficulty intubating the trachea, or both” [9]. In contrast, the Canadian Airway Focus Group (CAFG) defines difficult intubation as “a situation in which a physician anticipates or encounters difficulty with, any or all of, facemask ventilation, direct or indirect ventilation (e.g., videolaryngoscopy), tracheal intubation, use of an epiglottal device, or requiring surgical airway access” [10].

The DAS guidelines, updated periodically, are an essential management tool for doctors and medics not only in the UK but around the world [11]. They emphasize planning, recognition and detailed airway management especially in the setting of difficult airways. Included are key elements for safe instrumented airway management, patient oxygenation, the RSI (Rapid Sequence Induction) procedure or the VORTEX acronym- for effective patient airway management. Difficult laryngoscopy is defined as a Cormack-Lehane scale 3 or 4. The frequency of failed difficult intubation and its evolution to a CICO scenario (Can’t Intubate, Can’t Oxygenate) is reported 1 in 180,000 cases the according to DAS, with only 25% of these reported as difficult airway situations only [12]. It is interesting to note that about one-third of the complications reported to NAP4 are related to anesthesiologist error in assessing difficult intubation, related to anatomical pathologies involving the neck and head of patients [13]. In the study by AliA et al. among 250 patients with maxillofacial problems, significant problems in airway management were reported in 21.2% of cases and difficult intubation in 6.4%, while problems during ventilation with a face mask occurred in 9.6% of cases [14]. In addition, the algorithm for the difficult intubation procedure includes equipment items such as soft and rigid guides or videolaryngoscopes. They significantly facilitate visualization and the proper assessment of the epiglottis, and their use has shown a higher percentage of correct placement of the endotracheal tube and intubation success rate with a decrease in intubation duration especially in pediatric patients. In a study involving more than 5000 children, the success rate of the first intubation attempt was higher compared to standard laryngoscopy, sequentially from 20.0 to 94.1% for the video laryngoscope and from 37.6 to 82.8% for the classic method [15].

### 1.2. Standard Forms of Airway Assessment and Their Predictive Value

There are numerous algorithms and systems for assessing a patient’s airway. The sensitivity and specificity of these tests vary widely and according to EBM a single test cannot effectively predict difficult intubation or patient ventilation problems [16]. Also, the LEMON scale, the Wilson risk test, the hyomental distance or the Patill test are also applicable and used when managing and predicting risk in difficult airways. The sensitivity and specificity of the aforementioned tools are still under investigation. A paper by Zhihen Wang et al. [17] presented a review of articles on difficult intubation risk assessment. The meta-analysis examined 11 methods for predicting difficult tracheal intubation, and included a total of 686,089 patients. It was found that the modified mallampati test showed a pooled sensitivity of 0.39, thyromental distance had a pooled sensitivity of 0.38, and for the upper lip bite test the pooled sensitivity was 0.52, while scales such as LEMON and Wilson’s risk score showed pooled sensitivities of 0.58 and 0.42, respectively. At the same time, studies analyzing the use of ultrasound in airway evaluation indicate their superiority over classical methods, and the much better sensitivity and specificity of these methods in detecting difficult airways. The authors concluded that future research should focus on the integration of advanced technologies such as artificial intelligence and take advantage of its deep learning capabilities in real time.

Similar observations are shown in a systematic review by RothD et al. [18], in which for difficult laryngoscopy, the summary sensitivity ranged from 0.22 for the mouth opening test to 0.67 for upper lip bite test and the summary specificity ranged from 0.80 for modified Mallampati test to 0.95 for Wilson risk score. The upper lip bite test provided the highest sensitivity among all the tests predicting difficult laryngoscopy. For difficult tracheal intubation, summary sensitivity ranged from 0.24 for thyromental distance to 0.51 for the modified Mallampati test and the summary specificity ranged from 0.87 for modified Mallampati test to 0.93 for mouth opening test. The modified Mallampati provided the highest sensitivity for difficult tracheal intubation prediction compared to the other tests. For difficult face mask ventilation, only estimated summary sensitivity (0.17) and specificity (0.90) for the modified Mallampati test were calculated.

Screening tests are supposed to have high sensitivities; however, all investigated index tests had low sensitivities with high variability. Moreover, their specificities were noticeable higher than the sensitivities across all tests. In other words, they are not good screening tests. As we do not have a precise tool for difficult airway prognostics, artificial intelligence may be at hand.

## 2. Methods

This article explores the role of AI in predicting difficult airways, focusing on machine learning models, deep learning applications, and the integration of AI in clinical practice. We used Pubmed and Researchgate for searching for articles. We searched for descriptions of AI-based models used in a bedside manner for the prediction of difficult intubation or difficult airways. There was no time restriction, as we searched for the first case of a clinically useful AI-based model and then searched for data until 2024. Only bedside AI-enhanced systems were taken into consideration.

### Artificial Intelligence in Difficult Airway Prognostics

In 2016, Caudet G.L. et al. [19] created one of the first AI-based models for difficult airway prediction. It was based on analysis data (photos, videos and ground truth data) obtained from 970 patients. They developed statistical face models from our database to automate facial morphology parametrization for predicting difficult intubation. Using random forest, we ranked the most discriminative morphological features and trained a classifier. We compare a class-prior threshold tuning method with two others that optimize thresholds to address the imbalanced nature of the data. Their model achieved AUC = 77.9%, being comparable with state-of-the-art medical diagnosis, and thus being applicable for real-world difficult intubation prediction. As the authors mentioned, they created the first fully automatic difficult intubation detection system that does not require any invasive procedure, which may be useful in clinical settings.

In 2021, Hayasaka T et al. [20] created an AI model using CNN to predict difficult intubation. A total of 16 different facial images were obtained, and classified by anesthesiologists as ”easy/difficult”. An AI classification model was created using deep learning by linking the patient’s facial image and the intubation difficulty. Their model’s accuracy was 80.5%; the sensitivity was 81.8%; the specificity was 83.3%; the AUCwas 0.864; and the 95% confidence intervalwas [0.731–0.969]. Receiver operating characteristic curves were generated for both actual intubation difficulty and the AI model, with sensitivity, specificity, and AUC calculated, using the median AUC as the result. Class activation heat maps focused on the neck area; the AI model identified facial contours to predict intubation difficulty. The AI model they developed may be useful in future for medical staff both in emergency situations or under elective anesthesia.

Also in the same year, Tavolara T.E. et al. [21] created a model, that was based on an analysis of frontal face images using CNN. They developed an ensemble of convolutional neural networks that uses a celebrity facial image database to learn robust features from various face regions. This ensemble extracts features from patient images (*n* = 152), which are then classified by an attention-based multiple-instance learning model. A majority voting system determines whether a patient is classified as difficult or easy to intubate. The proposed method resulted in AUC = 0.7105, surpassing bedside tests. The authors stated that the proposed model may operate at high sensitivity and low specificity (0.9079 and 0.4474, respectively) or low sensitivity and high specificity (0.3684 and 0.9605, respectively). Side facial images could enhance the performance of the proposed model. This model is believed be crucial in advancing deep learning techniques where frontal facial features are key.

In 2022, Yamanaka S., et al. [22] created v AI-based models using data from 10,741 intubations, out of which 543 were considered difficult intubations. As a reference model, the modified LEMON criteria were used. From all models used, a logistic regression model with elastic-net, random forest, gradient boosting decision tree, XGBoost, and an ensemble model provided better results than the reference model.

In 2023, Wang G. et al. [23] created, based on 1000 patient observations, a fully-automatic semi-supervised deep learning model for difficult airway assessment. The model used photographs obtained from nine different viewpoints. The dataset of collected images was partitioned into training and testing subsets in an 8:2 ratio. A semi-supervised deep learning approach was employed to develop and evaluate an AI model for predicting difficult airways. Model training utilized only 30% of the labeled training data, while the remaining 70% were incorporated without labels. The model’s performance was assessed using accuracy, sensitivity, specificity, F1-score, and area under the receiver operating characteristic (ROC) curve (AUC) of 90.00%, 89.58%, 90.13%, 81.13%, and 0.9435, respectively. The author’s findings indicate that the semi-supervised deep learning model, trained with only 30% of labeled samples, achieves a performance comparable to that of a fully supervised model while significantly reducing the cost of data labeling. This approach effectively balances model performance and resource efficiency. Furthermore, the performance of the semi-supervised model closely approximates that of human experts. To the best of the author’s knowledge, this study is the first to implement a semi-supervised deep learning approach for the simultaneous prediction of both mask ventilation and intubation difficulties. The proposed AI-driven image analysis system has the potential to serve as a valuable tool for identifying patients at risk of difficult airway conditions.

In the same year, Kim J.H. et al. [24] created a machine-learning based model based on the analysis of difficult airway predictors in a population of 1677 anesthetized patients. The variables were age, sex, height, weight, body mass index, neck circumference, and thyromental distance. The dataset was randomly stratified into a training set (80%) and a test set (20%), ensuring an equal distribution of difficult laryngoscopy cases. Five machine learning algorithms—logistic regression, multilayer perceptron, random forest, extreme gradient boosting, and light gradient boosting machine—were utilized for model training. The predictive models were subsequently evaluated using the test set for validation. As a result a model based on random forest was best in predicting difficult laryngoscopy with an area under receiver operating charesteristic curve (AUROC) = 0.79 [95% confidence interval: 0.72–0.86]. The authors claimed that adding more data, a new variable and a combination of models can result in even better results.

In 2024, Xia M. et al. [25] created a model using a Light Gradient-Boosting Machine algorithm to predict difficult videolaryngoscopy. They used data such as baseline characteristics, medical history, bedside examination and seven facial images obtained from 5849 patients. ResNet-18 was employed for image recognition and feature extraction. Various machine learning algorithms were implemented to construct predictive models. The model mentioned above showed the highest area under the curve (95%CI) of 0.779 (0.733–0.825) with a sensitivity (95%CI) of 0.757 (0.650–0.845) and specificity (95%CI) of 0.721 (0.626–0.794) in the test set, which was a significantly better outcome than the bedside examination and multivariate scores.

Also in 2024, Kim J.H. et al. [26] created a deep learning model based on an analysis of 18,163 pictures from 3053 patients, which were obtained from frontal, lateral, frontal neck extension, and open mouth views. A deep learning model based on the EfficientNet-B5 architecture was developed for predicting difficult direct laryngoscopy (DDL), integrating image view information through multitask learning. To balance the dataset, under-sampling was applied to achieve a 1:1 ratio of DDL to non-DDL images. The model was trained and validated using a dataset comprising 6616 images from 1283 patients. Performance evaluation demonstrated a receiver operating characteristic area under the curve (AUC) ranging from 0.81 to 0.88 and an F1-score between 0.72 and 0.81 for DDL prediction. Incorporating image view information enhanced model performance. Additionally, gradient-weighted class activation mapping identified neck and chin features in frontal and lateral views as key contributors to DDL prediction. The model effectively predicted difficult direct laryngoscopy and requires small numer of pictures taken with a smartphone.

## 3. Discussion

The integration of artificial intelligence (AI) in healthcare has gained substantial importance in recent years, offering numerous benefits. A review of the existing studies indicates that AI has the potential to support healthcare professionals by automating repetitive tasks, improving medication accuracy, enhancing clinical decision-making, and facilitating interdisciplinary collaboration. Additionally, AI has demonstrated significant advantages in medical imaging, disease diagnosis and treatment, virtual health assistance, and drug development.

Despite these benefits, challenges remain in the implementation of AI in healthcare. The accessing and utilizing of patient data are hindered by privacy concerns, while other barriers include limited patient awareness, technological constraints, and issues related to professional liability. Ethical considerations, particularly regarding data privacy and algorithmic bias, are among the most critical challenges. Ensuring the protection of patient information and preventing the reinforcement of social biases is essential for the responsible and equitable deployment of AI in healthcare. Furthermore, limited public understanding of AI may contribute to skepticism and resistance to its adoption.

Nevertheless, AI presents significant opportunities, such as improving collaboration among healthcare providers, advancing diagnostic capabilities, enhancing patient monitoring, and supporting virtual health services. By analyzing large volumes of medical data, AI can aid clinicians in making more informed decisions, ultimately leading to better patient outcomes and more efficient resource allocation. AI has the potential to revolutionize healthcare by enabling more precise diagnoses, personalized treatment strategies, and optimized clinical workflows [27,28]. Regarding AI-based bedside algorithms, it is clear that devices will be developed in the near future to assist physicians in assessing the airway at the preoperative anesthesia consultation stage. It is also possible to use the data collected during this evaluation in the preparation of robotic intubation systems, such as the Kepler Intubation System (KIS) [29]. This system was developed to relieve the anesthesiologist in the operating room. While, thus far, it is too large and too imprecise, and, in the authors’ words, “problematic to use” to be widely introduced into clinical practice [30], the future creation of a system based on data exchange between artificial intelligence-based airway assessment systems and an intubation robot will represent a breakthrough in immediate perioperative patient care.

Future research should focus on creating an artificial intelligence-based airway assessment system that will be realistically implemented in clinical practice and will learn as it operates, collecting data and improving its performance based on them. This system should be able to transmit data to the hospital’s information system, which in turn will transmit it to robotic systems working in the operating theater, enabling it to prepare for potential difficult intubation.

## 4. Conclusions

Artificial Intelligence-based algorithms are creating a new quality in predicting difficult airways. In the available studies, they show high sensitivity and specificity, with which they significantly outperform classical tests based on complex scales and indices, matching and even surpassing the abilities of experienced anesthesiologists. The future of difficult airway assessment is likely to be systems based on handheld tablets that take pictures of patients’ faces during preoperative anesthesia consultation and assess the risk of airway management difficulties, including difficult intubation. Perhaps in the future a system will be developed that, in addition to assessing the risk, will also indicate what instruments for securing the airway should be prepared and what problems the physician securing the airway may face and may transfer assessment data to robotic intubation systems in the operating theatre.
